# The complexity of producing and interpreting dietary vitamin A statistics

**DOI:** 10.1016/j.jfca.2021.103926

**Published:** 2021-07

**Authors:** Ana Moltedo, Cristina Álvarez-Sánchez, Fernanda Grande, U. Ruth Charrondiere

**Affiliations:** a Statistics Division, Food and Agriculture Organization of the United Nations, Viale delle Terme di Caracalla, 00153, Rome, Italy; b Food and Nutrition Division, Food and Agriculture Organization of the United Nations, Viale delle Terme di Caracalla, 00153, Rome, Italy

**Keywords:** Household consumption and expenditure surveys, HCES, Vitamin A, Retinol Equivalent, Retinol Activity Equivalent, Carotenoids, Dietary assessment

## Abstract

Producing, reporting, and interpreting vitamin A statistics present multiple challenges largely attributable to the systems of equivalence used to convert pro-vitamin A carotenoids into retinol equivalents, and to the criteria used by institutions to set recommendations. This study describes the information on total vitamin A, retinol and provitamin A carotenoids available in 90 food composition tables/databases (FCTs/FCDBs). It also evaluates the effect of the definition of vitamin A intake (Retinol Equivalents [RE] or Retinol Activity Equivalents [RAE]) and the source of requirements on the potential contribution of dietary intake to the population’s requirements. We found that 43 percent of the FCTs/FCDBs reviewed, many of them from high-income countries, do not provide total vitamin A or sufficient information for computing it, or present inconsistencies between the metadata and the published values; 9 percent publish total vitamin A in RE and RAE; and 28 percent provide information on retinol and provitamin A carotenoids that enables calculating total vitamin A in both definitions.

Vitamin A adequacy ratios are lowest when the consumption unit is RAE and the source of requirements is the US Health and Medicine Division. When the consumption definition is RE, adequacy ratios are higher using FAO/WHO than EFSA requirements. It is imperative to reach consensus on the system of conversion of provitamin A carotenoids into retinol equivalents.

## Highlights

• 38 of 90 food composition tables reviewed report total vitamin A values poorly.
• Only 9 tables provide total vitamin A values expressed in both RE and RAE.
• 25 tables provide enough information to calculate total vitamin A in RE and RAE.
• Consensus on the conversion of pro-vitamin A carotenoids to retinol is needed.
• Vitamin A adequacy ratios vary with the unit of intake and source of requirements.

## 1 Introduction

Vitamin A statistics (intake/supply and requirements) are used worldwide by professionals in governments, academia, health services and the food industry, for a broad set of purposes such as assessing the level of inadequacy by sex-age groups, evaluating the potentiality of the food supply to meet nutrition needs in a country, identifying fortification vehicles, and nutrition-sensitive programming in agriculture. However, producing, reporting, and interpreting vitamin A statistics present multiple challenges (Melse-Boonstra et al., 2017). Analytical difficulties exist when generating and presenting accurate compositional data on vitamin A and provitamin A components. For instance, diverse analytical and saponification methods generate significantly different values, genetics and feed influence vitamin A content hugely, and carotenoid values are rarely available and need to be estimated. These types of challenges have been well described elsewhere (Mercadante et al., 2017; Rodriguez-Amaya, 2010; Rodriguez-Amaya et al., 2008). Furthermore, different systems of equivalence exist and are applied to convert provitamin A carotenoids into vitamin A (Grande et al., 2016). In addition, vitamin A recommended daily intakes (RDI) vary significantly according to the criteria used by institutions to set these recommendations. Last but not least, often producers and users of dietary vitamin A consumption statistics are unaware of the existence of different systems of equivalences and of the differences between the RDI published by national and international institutions combining them indistinctly (e.g., Harika et al., 2017; López-Sobaler et al., 2017). Consequently, nutrition and health policies and programs may be based on inaccurate vitamin A statistics and indicators –– such as vitamin A inadequacy –– and, thus, expected outcomes would be misguided and perhaps not achieved.

### 1.1 Definitions of total vitamin A intake

Vitamin A is available in foods in two forms: preformed vitamin A (retinol) and provitamin A carotenoids. Retinol is found in animal-source foods, such as meat (especially liver), dairy products, eggs and fish. The major source of carotenoids is plant food, primarily orange and yellow vegetables and fruits, and dark green leafy vegetables, as well as red palm oil. The most well-known provitamin A carotenoid is beta-carotene; other provitamin A carotenoids include alpha-carotene and beta-cryptoxanthin. Given that carotenoids can be converted in the body into vitamin A, systems have been created to estimate their retinol equivalence so that the content of vitamin A between different foods (particularly between animal- and plant-source foods) can be comparable and so that dietary vitamin A can be expressed on a common basis.

In 1967, FAO and WHO (FAO and WHO, 2004) defined a system of equivalence that expresses total vitamin A in μg of retinol equivalents (RE), where total vitamin A =1 μg all-*trans* retinol + 1/6 μg beta-carotene + 1/12 μg (other provitamin A carotenoids).

Underlying studies of the United States Institute of Medicine (2006) report showed evidence that the efficiency in absorption of dietary provitamin A carotenoids is lower than what was traditionally thought (Institute of Medicine, 2006). Thus, since 2001, the United States (US) Health and Medicine Division (HMD) recommends a different system of equivalence that expresses vitamin A in μg of retinol activity equivalents (RAE), where total Vitamin A = 1 all-trans μg retinol + 1/12 μg beta-carotene + 1/24 μg (other provitamin A carotenoids). In other words, when vitamin A in foods is expressed as RAE, the provitamin A activity is considered half the activity than previously defined by FAO and WHO.

In some cases, other provitamin A activity conversion factors have been used (Bogard et al., 2015; Dube et al., 2018).

Bioconversion of carotenoids to vitamin A in the human body is affected by several factors including carotenoid content in foods, meal composition, genetic polymorphisms, the amount of vitamin A stored in the body, and an individual’s health status (Olmedilla-Alonso et al., 2020; Tang, 2010; Tanumihardjo et al., 2010). Additionally, in contrast to the conversion factors currently being used, recent studies showed that alpha-carotene and especially beta-cryptoxanthin have a greater apparent bioavailability than beta-carotene (Burri et al., 2011; Olmedilla-Alonso et al., 2020).

In summary, not only there is no scientific agreement on a single system of equivalence to express vitamin A in foods but we could also expect future changes on the current existing ones (Dias et al., 2018), thereby creating confusion among producers and users of dietary vitamin A statistics.

### 1.2 Sources of recommended daily intake

International and national expert committees establish recommended daily intake, also called dietary nutrient reference values, for specific sex-age groups and for pregnant/lactating women. The key nutrient reference value for estimating the level of inadequate dietary intake within a group is the Estimated Average Requirements (EAR) (also called Average Requirements [AR]) (US Institute of Medicine, 2000). The EAR is the average daily nutrient intake level to meet the requirement of half the population in a group of healthy individuals of the same sex and similar age (US Institute of Medicine, 2000). FAO and WHO (FAO and WHO, 2004) and the European Food Safety Authority (EFSA) (EFSA Panel on Dietetic Products Nutrition and Allergies, 2015) published EARs and ARs for vitamin A, respectively, keeping RE as the expression of intake. FAO and WHO nutrient requirements were set for populations worldwide, and for vitamin A were defined as the minimum average daily intake to prevent xerophthalmia in the absence of clinical or sub-clinical infection. EFSA nutrient requirements were defined for populations in European countries to allow for adequate growth and other dependent functions, and to maintain an acceptable total body pool of vitamin A. Even though nutrient requirements should be developed on a country basis only few countries, such as India (National Institute of Nutrition, 2010), the United States of America (US) and Canada (HMD, 2016), have developed their own guidelines for vitamin A requirements. A few other countries adopted recommendations established by another country agency. This is the case for Colombia, which adopted the US HMD recommendations (Ministerio de Salud y Protección Social de Colombia, 2016). The risk of using vitamin A requirements for populations other than those they are intended for, is that there may be a gross over or underestimation of vitamin A inadequacy. The US HMD published EARs of vitamin A, which assure adequate liver vitamin A stores, and keeps RAE as the expression of intake (HMD, 2016). Countries and researchers largely rely on the FAO/WHO, EFSA and US requirements (O’Neill et al., 2020).

### 1.3 Lack of harmonization in the definition of total vitamin A in Food Composition Tables and Databases

The lack of international consensus on one system of equivalence to express vitamin A in foods is reflected in Food Composition Tables and Databases (FCTs/FCDBs). It has been a common practice to publish FCTs/FCDBs expressing total vitamin A in foods in either RE or RAE; however, a few up-to-date versions of FCTs/FCDBs include total vitamin A in both RE and RAE (e.g. National Institute for Public Health and the Environment, 2019; Universidade de São Paulo Food Research Center, 2020; Vincent et al., 2020). In some cases, FCTs/FCDBs present the content of retinol and provitamin A carotenoids (e.g. Indian FCTs (Longvah et al., 2017); United States FCD (U.S. Department of Agriculture Agricultural Research Service, 2019) making it possible for researchers to calculate total vitamin A in foods using the definition of interest.

### 1.4 Use of food consumption data from household consumption and expenditure surveys

Due to the scarcity of nationally and/or regionally-representative individual dietary intake surveys, even in high-income countries (Rippin et al., 2018), Household Consumption and Expenditure Surveys (HCES) are used for dietary assessment, including vitamin A inadequacy, despite their limitations (e.g. Gilbert et al., 2019; Troubat et al., 2020). HCES are multipurpose surveys, conducted in a large number of countries every two to ten years. Even though, they are not specifically designed for food security analyses, they collect food quantities consumed and/or acquired by households. Depending on their design, and how the food data are collected and analyzed, dietary nutrient statistics could underestimate, or, more often, overestimate actual nutrient intake (Karageorgou et al., 2018; Zezza et al., 2017). In contrast to individual dietary intake surveys that collect information on individuals’ food intake (i.e. food ingested), HCES collect information on food “apparently” consumed at the household level. Therefore, herein, we use the term “vitamin A consumption” instead of “vitamin A intake”.

The objectives of the current analysis were: (1) to evaluate the effect on the ratio vitamin A consumption/requirements, using requirements published by FAO and WHO, EFSA, and US HMD, and consumption expressed in μg of RE or RAE according to the source of requirements; and (2) to identify the vitamin A information (i.e., vitamin A, retinol, beta-carotene, beta-carotene equivalents, alpha-carotene and beta-cryptoxanthin) available in national and regional FCTs/FCDBs.

## 2 Materials and methods

### 2.1 Impact of the definition of vitamin A intake and of the source of requirements

To address the first objective, we analyzed food consumption data collected between 2003 and 2010 in five HCES. The HCES were: the Bangladesh Household Income and Expenditure Survey 2010 (Bangladesh Bureau of Statistics, 2010), the Cambodia Socio Economic Survey 2009 (Cambodia National Institute of Statistics, 2009), the Tanzania Household Budget Survey 2007 (Tanzania National Bureau of Statistics, 2007), the Uganda National Household Survey 2005/06 (Uganda Bureau of Statistics, 2006) and the Vietnam Household Living Standard Survey 2010 (General Statistics Office of Vietnam, 2010). However, the results are shown under the pseudonyms *Country A*, *B*, *C*, *D* and *E*, and not by the names of the surveys, because we do not have explicit permission from the data owners to disclose the derived statistics associated to the surveys. Moreover, the choice of these surveys was not made with the intention to analyze and interpret the vitamin A consumption levels in specific countries, but to be used as examples. Therefore, the fact that the statistics are not associated to the surveys is not regarded as a limitation. The criteria used for the selection of the surveys included data availability and comprehensive coverage of food modules characteristics in terms of reference period, type of food module (i.e., acquisition/consumption), number of foods, etc. Table 1 presents some of the characteristics of the food modules and the samples in the five HCES.

**Table 1:** Characteristics of the food modules and the samples in the five HCES datasets.

Food module	Country A		Country B	Country C	Country D	Country E
Module type^a^	Consumption and acquisition		Consumption and acquisition	Acquisition	Consumption and acquisition	Consumption
Reference period	Last 7 days		Last month	Last month	Last 12 months	Last 14 or 7 days
Food data collection method	Recall with a predefined list		Open diary	Open diary	Recall with a predefined list	Diary/recall with a predefined list
Total number of food items reported	56		194	222	52	138
Households reporting consumption of at least one poorly defined food^b^	27 %		86 %	54 %	93 %	72 %
Percentage of poorly defined foods in total foods declared by households	2.9 %		2.8 %	4.8 %	24.1 %	0.4 %
No. HHs Sampled & Represented						
National	7419 & 5,229,645	11,970 & 2,938,408		10,421 & 8,060,230	9398 & 22,275,698	12,240 & 33,028,014

No. HHs, number of households.

a “Consumption and acquisition” refers to a survey collecting data on food acquisition from purchases and on food consumption from other sources such as own production or received in kind. “Consumption” refers to a survey collecting data on food consumption, considering all food sources. “Acquisition” refers to a survey collecting data on food acquisition considering all food sources.

b Poorly defined foods cannot be matched to food items in an FCT/FCDB because their ingredients are not known. Therefore, the nutrient content in poorly defined foods is estimated applying median (at region, urban or rural area, and income quintile level) at-home nutrient unit values to the amount spent to acquire these foods.

For each of the five countries, we estimated vitamin A adequacy ratios (ratios of consumption to requirements, known as Nutrient Adequacy Ratios [NARs]) for five population groups: the whole country (i.e., national level), urban areas, rural areas, lowest income quintile (poorest) and highest income quintile (richest); using the ADePT-Food Security Module (ADePT-FSM) tool version 3 (FAO, 2021). Even though the NAR is not a measure of the level of nutrient inadequacy in a population –– the inadequacy measure depends not only on the average intake but also on the shape of and variability of the usual intake distribution –– it is useful for assessing the relative size of nutrient intake in relation to requirements in a group. It has been used for informing policies and programs (e.g. Marivoet and Ulimwengu, 2018) and to build the Mean Adequacy Ratio indicator (e.g. Akter et al., 2021).

We standardized reported food quantities into grams or liters; and quantities in liters were then converted to grams using density factors (FAO/INFOODS, 2012a). Then, we adjusted food quantities in grams for edible portions and matched the survey foods with raw foods in FCTs/FCDBs, preferably designed for the country, following FAO/INFOODS (2012b) food matching guidelines. When necessary, we complemented with other two FCTs/FCDBs. We compiled information on retinol, beta-carotene, alpha-carotene, beta-cryptoxanthin, and beta-carotene equivalents according to information available in the FCTs/FCDBs. For each food, we calculated its total vitamin A content, expressed in μg of RE and RAE, from information on retinol and provitamin A carotenoids. The five FCTs/FCDBs used were: A Food Composition Table for Central and Eastern Uganda (Hotz et al., 2012), Food Composition Table for Bangladesh (Shaheen et al., 2013), Food Data Central (U.S. Department of Agriculture Agricultural Research Service, 2019), SMILING D3.5-a Food composition table for Cambodia (Ministry of Agriculture Forestry and Fisheries of Cambodia, Cambodia, M. of H. of, Wageningen University of Netherlands, 2013), and the Vietnamese Food Composition Table (Ministry of Health, 2007). The two FCTs/FCDBs used for remaining missing values were the ASEAN Food Composition Database (Mahidol University, 2014) and the Tanzania Food Composition Tables (Muhimbili University of Health and Allied Sciences, Tanzania Food and Nutrition Center, Harvard School of Public Health, 2008). All five HCES datasets contained poorly defined foods (such as “dinner in restaurant”, “lunch at work” and “meal at a street vendor”) in their food module list without reporting food quantities for them. Therefore, the amount of vitamin A contributed by these foods was not estimated using a food matching approach but through the ADePT-FSM tool, using the respective food monetary values combined with median at-home vitamin A unit monetary values (Moltedo et al., 2018). Then, for each household, we summed the vitamin A content in foods obtained from the food matching approach and from the monetary value approach.

The HCES analyzed collected food quantities consumed and/or acquired by households without information on intra household food distribution (as is common in this type of survey). Therefore, to adjust for between-household differences in vitamin A consumption due to differences in household size (i.e., the number of household members), we divided each household’s total vitamin A consumption by the household’s corresponding number of members. Then, to convert vitamin A consumption on a daily basis, we divided the per capita values by the number of days corresponding to the food module reference period. By performing the average of all households’ daily per capita vitamin A consumption random errors are cancelled out and we assume that the average consumption is an unbiased estimate of the mean of the distribution of usual vitamin A consumption. We used weights (household weight times the number of household members) to infer the average consumption estimate at the population level.

The levels of intake represented by the EAR are published for groups of healthy individuals of the same sex and similar age; however, individuals belonging to different sex and age groups composed the five population groups analyzed in this study. Therefore, the EAR of each population group under analysis was computed as the weighted average of the EAR in each sex-age group (using the proportion of household members in each sex-age group as weights). Table 2 presents the sex-age EAR by source of requirements. The HCES data analyzed did not collect information on the physiological status of women; therefore, one of the limitations of this analysis is that all females were assumed to be non-pregnant and non-lactating. The main differences between the sources of requirements are the unit of intake and/or the criteria to set the appropriate level of usual intake. Therefore, we estimated three levels of daily per capita requirements (i.e., EARs) for each of the five populations (in μg).

**Table 2:** Vitamin A Estimated Average Requirements, used in the analysis, by sex-age group and source of requirements.

US HMD		
Sex	Age	μg in RAE
Male	0–11 month	450
Male	1–3 year	210
Male	4–8 year	275
Male	9–13 year	445
Male	14–18 year	630
Male	More than 18 year	625
Female	0–11 month	450
Female	1–3 year	210
Female	4–8 year	275
Female	9–13 year	420
Female	14–18 year	485
Female	More than 18 year	500

FAO/WHO		
Sex	Age	μg in RE
Male	0–11 month	185
Male	1–6 year	200
Male	7–9 year	250
Male	10–18 year	365
Male	More than 18 year	300
Female	0–11 month	185
Female	1–6 year	200
Female	7–9 year	250
Female	10–18 year	365
Female	19–65 year	270
Female	More than 65 year	300

EFSA		
Sex	Age	μg in RE
Male	0–11 month	270
Male	1–3 year	205
Male	4–6 year	245
Male	7–10 year	320
Male	11–14 year	480
Male	15–17 year	580
Male	More than 17 year	570
Female	0–11 month	190
Female	1–3 year	205
Female	4–6 year	245
Female	7–10 year	320
Female	11–14 year	480
Female	More than 14 year	490

We defined three models to derive the adequacy ratios in each population: (a) RAE-HMD, where vitamin A consumption in μg of RAE is compared to the US HMD vitamin A requirements; (b) RE-FAO/WHO, where vitamin A consumption in μg of RE is compared to FAO and WHO vitamin A requirements; and (c) RE-EFSA, where vitamin A consumption in μg of RE is compared to EFSA vitamin A requirements.

Vitamin A requirements published by US HMD are in general higher than those published by EFSA, which at the same time are higher than those published by FAO/WHO. As the definition of RAE considers a lower bio-availability of provitamin A carotenoids in plant foods, consumption expressed in μg of RE is always higher than expressed in RAE. Therefore, it is expected that the combination consumption in RAE with requirements from US HMD would produce the lowest adequacy ratio, the combination consumption in RE with requirements from FAO/WHO would produce the highest adequacy ratio, and the combination consumption in RE with requirements from EFSA would be in the middle. The question was how much the adequacy ratios would differ one from each other.

### 2.2 Review of Food Composition Tables and Databases for the type of vitamin A information available

To achieve the second objective, we considered the most recent versions of FCTs/FCDBs published by national or regional institutions and available online in English, French or Spanish. When the online FCT/FCDB was not available for public use, we contacted the authors for further information and/or clarification. Just a small number of the FCTs/FCDBs were only in print. We reviewed 90 FCTs/FCDBs and extracted information on an Excel sheet on the availability of total vitamin A, retinol, beta-carotene, beta-carotene equivalents, alpha-carotene and beta-cryptoxanthin values as per INFOODS’ components identifiers, called Tagnames. Table 3 presents a detailed definition of each INFOODS Tagname prepared from Klensin et al. (1989) and updated in 2007 (FAO/INFOODS, 2021).

**Table 3:** INFOODS Tagnames for vitamin A, retinol and provitamin A carotenoids.

INFOODS Tagname	Definition	Unit
<VITA_RAE>	Calculated total vitamin A activity expressed in μg retinol activity equivalent (RAE) = μg retinol + 1/12 μg beta-carotene + 1/24 μg other pro-vitamin A carotenoids (or μg RAE = μg retinol + 1/12 μg beta-carotene equivalent)	μg
<VITA>	Calculated total vitamin A activity expressed in μg retinol equivalent (RE) = μg retinol + 1/6 μg beta-carotene + 1/12 μg other pro-vitamin A carotenoids (or μg RE = μg retinol + 1/6 μg beta-carotene equivalent)	μg
<CARTA>	Alpha-carotene. All-trans alpha-carotene only	μg
<CARTB>	Beta-carotene. All-trans beta-carotene only	μg
<CRYPXB>	Beta-cryptoxanthin	μg
<CARTBEQ>	Beta-carotene equivalents = μg beta-carotene + 0.5 (μg alpha-carotene + μg beta-cryptoxanthin)	μg
<RETOL>	Retinol (synonyms of preformed vitamin A). All-trans retinol only	μg

First, we classified the FCTs/FCDBs into five geographical regions (Africa, Americas, Asia, Europe, and Oceania) according to the United Nations Standard Country or Area Codes for Statistical Use (M49 standard) classification (United Nations Statistics Division, 2020). Then, we classified them in one of nine groups, Group-1 is considered the most preferred scenario, which considers sufficient information on total vitamin A and its precursors and Group-9 the least preferred one, which does not provide enough information to estimate vitamin A in foods. The allocation into one of the nine groups was based on: a) the vitamin A information, i.e., unit of expression, definition and their concordance with the published values (for example, if total vitamin A was published in RE, or RAE, and there was also information on retinol and beta-carotene equivalents, we checked that the system of equivalence used was indeed that of RE or RAE, b) the associated metadata, and c) the coherence between the two. The groups were defined as follow:

• Group-1: FCTs/FCDBs that published total vitamin A in RE and RAE, plus retinol, beta-carotene, alpha-carotene and beta-cryptoxanthin.
• Group-2: FCTs/FCDBs that published total vitamin A in RE and RAE, plus retinol and beta-carotene equivalents.
• Group-3: FCTs/FCDBs that published only total vitamin A in RE and RAE.
• Group-4: FCTs/FCDBs that were not classified in any of the previous groups but published enough information to let the user calculate total vitamin A in RE and RAE. For instance, if an FCT/FCDB publishes total vitamin A in RAE and retinol, it is possible to estimate total vitamin A in RE. First, beta-carotene equivalents are estimated from vitamin A in RAE and retinol. Then, using beta-carotene equivalents and retinol it is possible to estimate total vitamin A in RE.
• Group-5: FCTs/FCDBs that published only total vitamin A in RE.
• Group-6: FCTs/FCDBs that published only total vitamin A in RAE.
• Group-7: FCTs/FCDBs that published total vitamin A in RE or RAE computed using only retinol and beta-carotene (not including other types of carotene).
• Group-8: FCTs/FCDBs that published total vitamin A but did not mention which system of equivalence was used, or there was an inconsistency between the Tagname and the system of equivalence described. For instance, an FCT/FCDB published vitamin A with the Tagname < VITA>, which corresponds to vitamin A in μg of RE, but the system of equivalence described corresponds to the Tagname < VITA_RAE>, which corresponds to vitamin A in μg of RAE.
• Group-9: FCTs/FCDBs that published total vitamin A using a system of equivalence different from RE and RAE (for example, μg of total vitamin A = μg of retinol + total carotenoids/6) or that did not provide enough information to let the user calculate total vitamin A.

## 3 Results

### 3.1 Impact of the definition of vitamin A intake and of the source of requirements

Table 4 presents the average vitamin A consumption in μg of RE and RAE and the difference between them in absolute (μg) and relative (%) terms by population group. The smallest difference in vitamin A consumption in absolute terms was 144 μg/capita/day (for the poorest population group in *Country D,* whose main contributors of carotenoids were vegetables with 1434 μg/capita/day of beta-carotene equivalents, not shown in the table) and the highest difference was 902 μg/capita/day (for rural areas in *Country A,* whose main contributors of carotenoids were roots and tubers with 7873 μg/capita/day of beta-carotene equivalents, not shown in the table). The average difference in vitamin A consumption between RE and RAE was 386 μg/capita/day. In relative terms, differences across the five population groups are similar, with a maximum difference of 6 percentage points in *Country D*. However, among countries the difference varies from 38 to 50 %, while the average difference was 44 %.

**Table 4:** Vitamin A consumption, in μg of RE and μg of RAE, in the five countries at national, urban and rural levels, and for the poorest and richest (based on income quintiles).

	Percentage of retinol in total Vitamin A, in RE	Percentage of retinol in total Vitamin A, in RAE	Average consumption^a^		Difference (μg RE – μg RAE)	Difference in percentage change
	(%)	(%)	μg RAE/capita/day	μg RE/capita/day	μg/capita/day	%
Country A						
National	2.2	4.3	888	1737	849	49
Poorest	0.7	1.4	655	1301	646	50
Richest	5.4	10.2	989	1875	886	47
Urban	5.0	9.5	627	1191	564	47
Rural	1.9	3.7	937	1839	902	49
Country B						
National	20.9	34.5	394	652	258	40
Poorest	19.2	32.2	267	447	180	40
Richest	21.9	35.9	642	1052	410	39
Urban	20.1	33.4	599	998	399	40
Rural	21.2	34.9	348	574	226	39
Country C						
National	3.6	6.7	552	1029	477	46
Poorest	1.8	3.4	525	1003	478	48
Richest	7.6	13.6	605	1088	483	44
Urban	5.7	10.4	358	658	300	46
Rural	3.2	6.0	618	1155	537	46
Country D						
National	17.8	29.9	285	480	195	41
Poorest	11.8	21.0	185	329	144	44
Richest	22.8	36.8	422	681	259	38
Urban	21.8	35.4	349	568	219	39
Rural	15.6	26.8	257	443	186	42
Country E						
National	7.2	13.5	244	456	212	46
Poorest	3.6	7.0	188	362	174	48
Richest	11.7	21.0	313	560	247	44
Urban	9.8	17.8	250	456	206	45
Rural	6.3	11.9	242	455	213	47

a Average daily per capita consumption (adjusted for the structure of the population using household’s weight times number of household members).

**Fig. 1:**
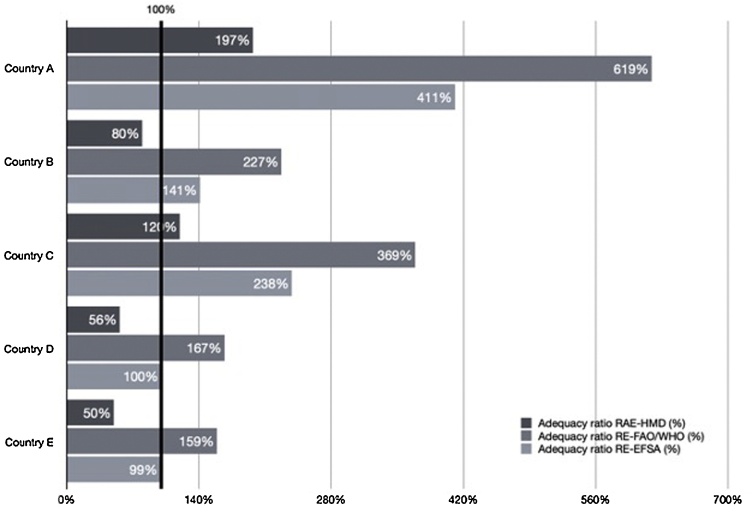
Vitamin A adequacy ratio for the five countries at national level. Figures notes: RAE-HMD: Vitamin A consumption is expressed in micrograms of Retinol Activity Equivalent and the source of requirements is US HMD. RE-EFSA: Vitamin A consumption is expressed in micrograms of Retinol Equivalent and the source of requirements is FAO/WHO. RE-FAO/WHO: Vitamin A consumption is expressed in micrograms of Retinol Equivalent and the source of requirements is EFSA.

As expected, among the three models, the RAE-HMD model had the lowest estimate for consumption and the highest estimate for requirements. Therefore, the vitamin A adequacy ratios using consumption in RAE and HMD requirements were invariably the lowest, while the ratios using consumption in RE and FAO/WHO requirements were the highest. In model RE-EFSA, the EFSA requirements have a criterion similar to HMD; however, the model estimates a higher level of consumption. This explains why the adequacy ratios using model RE-EFSA are in the middle between RAE-HMD and RE-FAO/WHO. This pattern is clearly visible in Fig. 1, which shows vitamin A adequacy ratios for the five countries at national level. In general, for the same population group, the model RAE-HMD produces adequacy ratios about three times lower than the RE-FAO/WHO model and two times lower than the RE-EFSA model.

Table 5 shows that using the model RE-FAO/WHO all population groups in all countries had an average consumption above their requirements. With the RE-EFSA model, most population groups in most countries had a ratio above 100. However, the RAE-HMD model identified two countries (D and E) in which the five population groups had consumption below their requirements. For instance, in *Country B*, according to the model RAE-HMD, the average vitamin A consumption of the poorest population was half their average requirements, while according to model RE-FAO/WHO their consumption surpassed their vitamin A requirements by 56 percentage points.

**Table 5:** Vitamin A consumption and adequacy ratio (%) in the five countries at national, urban and rural levels, and for the poorest and richest (based on income quintiles).

	RAE-HMD^a^			RE-FAO/WHO^b^			RE-EFSA^c^		
	Average consumption^d^ (μg RAE/capita/day)	Estimated Average Requirements^e^	Adequacy ratio (%)^f^	Average consumption^d^ (μg RE/capita/day)	Estimated Average Requirements^e^	Adequacy ratio (%)^f^	Average consumption^d^ (μg RE/capita/day)	Estimated Average Requirements^e^	Adequacy ratio (%)^f^
Country A									
National	888	450	197	1737	280	619	1737	423	411
Poorest	655	427	153	1301	273	476	1301	399	326
Richest	989	489	202	1875	291	645	1875	461	407
Urban	627	466	134	1191	284	420	1191	438	272
Rural	937	447	209	1839	280	657	1839	420	438
Country B									
National	394	492	80	652	287	227	652	463	141
Poorest	267	480	56	447	286	156	447	451	99
Richest	642	507	127	1052	287	366	1052	478	220
Urban	599	503	119	998	288	347	998	474	210
Rural	348	489	71	574	287	200	574	461	125
Country C									
National	552	460	120	1029	279	369	1029	432	238
Poorest	525	441	119	1003	274	366	1003	411	244
Richest	605	495	122	1088	284	383	1088	466	234
Urban	358	473	76	658	282	233	658	445	148
Rural	618	456	136	1155	278	415	1155	428	270
Country D									
National	285	509	56	480	287	167	480	480	100
Poorest	185	492	38	329	287	115	329	464	71
Richest	422	519	81	681	286	238	681	490	139
Urban	349	511	68	568	285	199	568	482	118
Rural	257	508	51	443	288	154	443	479	92
Country E									
National	244	486	50	456	286	159	456	458	99
Poorest	188	459	41	362	279	130	362	433	84
Richest	313	509	61	560	291	192	560	480	117
Urban	250	494	51	456	287	159	456	466	98
Rural	242	483	50	455	285	160	455	456	100

a Vitamin A consumption is expressed in micrograms of Retinol Activity Equivalent and the source of requirements is US HMD requirements.

b Vitamin A consumption is expressed in micrograms of Retinol Equivalent and the source of requirements is FAO/WHO requirements.

c Vitamin A consumption is expressed in micrograms of Retinol Equivalent and the source of requirements is EFSA requirements.

d Average daily per capita consumption (adjusted by the structure of the population using household’s weight times number of household members as weights).

e Average of the Estimated Average Requirements in each sex-age group (adjusted by the structure of the population using the proportion of individuals in each sex-age group as weights).

f Adequacy ratio was estimated as weighted average consumption divided by weighted average requirements.

### 3.2 Review of Food Composition Tables and Databases for the type of vitamin A information available

Out of the 90 FCTs/FCDBs reviewed, 30 % are from Europe, 24 % are from Asia, 22 % are from the Americas, 19 % are from Africa, and 4 % from Oceania (Table 6). From the 90 FCTs/FCDBs, 73 % were published or updated after 2009, 19 % between 2000 and 2009, and the remaining FCTs/FCDBs were published before 2000.

**Table 6:** Classification of 90 FCTs/FCDBs in groups according to availability of total vitamin A in RE and RAE, retinol and provitamin A carotenoids, and the possibility of calculating total vitamin A from retinol and provitamin A carotenoids.

Country	Publication- or update-referenced period^a^	Group 1^b^	Group 2^c^	Group 3^d^	Group 4^e^	Group 5^f^	Group 6^g^	Group 7^h^	Group 8^i^	Group 9^j^
All regions (*n [%]*)	–	4 (4 %)	3 (3 %)	2 (2 %)	25 (28 %)	4 (4 %)	2 (2 %)	12 (13 %)	24 (27 %)	14 (16 %)
Africa (*n [%]*)	–	1 (6 %)	1 (6 %)	1 (6 %)	5 (29 %)	0 (0 %)	0 (0 %)	1 (6 %)	5 (29 %)	3 (18 %)
Burkina Faso	2								X	
Ethiopia	1							X		
Gambia	3									X
Kenya	3		X							
Lesotho	2								X	
Malawi	3			X						
Mali	2								X	
Mozambique	3				X					
Nigeria	1				X					
South Africa	3				X					
Sudan	1									X
Tanzania	2								X	
Tunisia	2								X	
Uganda	3				X					
West Africa	3				X					
West Africa	3	X								
Zimbabwe	1									X
Asia (*n [%]*)	–	0 (0 %)	0 (0 %)	1 (5 %)	7 (32 %)	1 (5 %)	0 (0 %)	6 (27 %)	5 (23 %)	2 (9 %)
Armenia	3				X					
ASEAN	3							X		
Bahrain	3									X
Bangladesh	3				X					
Cambodia	3				X					
China	3					X				
India	3				X					
Indonesia	3				X					
Japan	3				X					
Korea, Republic of	3								X	
Korea, Republic of	3							X		
Laos	3								X	
Malaysia	1								X	
Nepal	3									X
Pakistan	2							X		
Philippines	1							X		
Singapore	3								X	
Thailand	2							X		
Thailand	3							X		
Turkey	3								X	
Vietnam	2				X					
Vietnam	3			X						
Europe (*n [%]*)	–	2 (7 %)	1 (4 %)	0 (0 %)	4 (15 %)	2 (7 %)	1 (4 %)	5 (19 %)	9 (33 %)	3 (11 %)
Austria	3					X				
Belgium	3							X		
Czech Republic	3								X	
Cyprus	3									X
Denmark	3								X	
England	3				X					
Estonia	3								X	
Faroe Islands	1							X		
Finland	3						X			
France	3							X		
German	3							X		
Greece	2									X
Greece	2									X
Iceland	3								X	
Italy	3				X					
Italy	3								X	
Netherlands	3	X								
Norway	3							X		
Portugal	3								X	
Serbia	3					X				
Slovak Republic	3				X					
Spain	3								X	
Sweden	3								X	
Switzerland	3		X							
United Kingdom	3				X					
FAO	3							X		
FAO	3	X								
Americas (*n [%]*)	–	1 (5 %)	0 (0 %)	0 (0 %)	7 (35 %)	1 (5 %)	1 (5 %)	0 (0 %)	4 (20 %)	6 (30 %)
Argentina	3									X
Bolivia	2									X
Brazil	2				X					
Brazil	3									X
Brazil	3								X	
Brazil	3	X								
Canada	3								X	
Central America	3						X			
Colombia	3								X	
Costa Rica	3									X
Ecuador	3									X
Latin America	3				X					
Mexico	3				X					
Peru	2									X
Peru	2								X	
Peru	3				X					
United States	3				X					
Uruguay	2					X				
Venezuela	2				X					
Ibero-America	3				X					
Oceania (*n [%]*)	–	0 (0 %)	1 (25 %)	0 (0 %)	2 (50 %)	0 (0 %)	0 (0 %)	0 (0 %)	1 (25 %)	0 (0 %)
Australia	3				X					
New Zealand	3				X					
Pacific Islands	2								X	
Pacific Islands	3		X							

a The number 1 refers to: prior to 2000, the number 2 refers to: between 2000 and 2009, and the number 3 refers to: after 2009.

b Group-1: FCTs/FCDBs published total vitamin A in RE and RAE, retinol and precursors of vitamin A.

c Group-2: FCTs/FCDBs published total vitamin A in RE and RAE, retinol and beta-carotene equivalents.

d Group-3: FCTs/FCDBs published total vitamin A in RE and RAE.

e Group-4: FCTs/FCDBs published enough information to let the user calculate total vitamin A in RE and RAE.

f Group-5: FCTs/FCDBs published only total vitamin A in RE.

g Group-6: FCTs/FCDBs published only total vitamin A in RAE.

h Group-7: FCTs/FCDBs published total vitamin A in RE or RAE computed using only retinol and beta-carotene (not beta-carotene equivalents).

i Group-8: FCTs/FCDBs published total vitamin A but there is no information on the system of equivalence used, or there was an inconsistency between the description of the system of equivalence and the unit of expression used.

j Group-9: FCTs/FCDBs published total vitamin A using a system of equivalence different from those widely used for establishing vitamin A requirement, or there was not enough information to estimate vitamin A.

The classification of the 90 FCTs/FCDBs into 9 groups according to the information on total vitamin A, retinol and provitamin A carotenoids provided is presented in Table 6. A more detailed description of the 90 FCTs/FCDBs is presented in Appendix A. Results show that only 9 % of the FCTs/FCDBs inform on vitamin A in RE and RAE (Group-1, Group-2 and Group-3), from which only 4 (4 %) were in the most preferred scenario (Group-1) including values for retinol, beta-carotene, alpha-carotene and beta-cryptoxanthin. A relevant number of FCTs/FCDBs (28 %) provide enough information to calculate total vitamin A in RE and RAE and were therefore classified in Group 4. A considerable number of FCTs/FCDBs (27 %) was categorized in Group-8, which means that the definition of vitamin A was not explicitly indicated or that there were inconsistencies between the Tagname, metadata and/or how vitamin A is expressed. In the least-preferred scenario (Group-9) there were 14 FCTs/FCDBs (16 %), meaning that they used a system of equivalence different than for RE and RAE or they did not provide enough information to calculate total vitamin A.

## 4 Discussion

### 4.1 Impact of the definition of vitamin A intake and of the source of requirements

We quantified the effect of the vitamin A definition in consumption estimates based on HCES data. Across the analyzed countries and groups, estimates of consumption in RE double the estimates in RAE; the lowest differences were observed in populations with more than 21 % and 35 % of preformed retinol of total vitamin A in RE and RAE, respectively, for which vitamin A consumption in RE was 1.6 higher than consumption in RAE.

We have demonstrated and quantified the impact of the criteria and source of vitamin A requirements on indicators based on a comparison between consumption and requirements like the NAR. Using HCES data, the model RAE-HMD always produced an adequacy ratio roughly three times lower than the RE-FAO/WHO model and two times lower than the RE-EFSA model. Differences in vitamin A adequacy ratios between the three models were independent from the percentage of preformed retinol in the group’s diet, for percentage values below 23 % and 37 % of preformed retinol of total vitamin A in RE and RAE, respectively. In the five countries using the three models, the poorest population groups had lower vitamin A adequacy ratios than the richest groups, whereas there was no pattern observed for rural *versus* urban adequacy ratio as it changed according to countries.

By looking at the adequacy ratio in a group, no inference can be made in terms of the prevalence of vitamin A inadequacy in the group, because there is no information on how vitamin A is distributed within the individuals in the group. When there is information on the prevalence of vitamin A inadequacy in a population (e.g., based on serum retinol analysis), the usefulness of the adequacy ratio is that it helps to identify whether the level of nutrient inadequacy could be mainly due to a low level of vitamin A intake or a high inequality in access to vitamin A within the group. However, the adequacy ratio based on each of the three models could be interpreted differently. For instance, for the poorest population in *Country B*, applying the RAE-HMD model, the adequacy ratio is 56 suggesting that low levels of vitamin A consumption might be the main cause of vitamin A inadequacy. On the contrary, according to the RE-FAO/WHO model the adequacy ratio for the same population is 156, suggesting that inequalities in accessing vitamin A might have an important role on vitamin A inadequacy.

Therefore, carefully considering the vitamin A consumption definition, and the source and criteria of requirements is of paramount importance when conducting dietary vitamin A analyses, as well as when comparing results between countries or from various sources. For example, in a study based on the 2012 Mexican National Health and Nutrition Survey, researchers estimated the prevalence of vitamin A deficiency (expressing intake in RAE and using the US HMD requirements) and pointed out that these prevalence values were higher than those measured with serum concentrations (Pedroza-Tobías et al., 2016). However, although they provided potential reasons for the divergence, they did not consider the differences in the criteria of requirements between the two measures. While the US HMD requirements are based on a level of intake that assures adequate liver vitamin A stores, the vitamin A deficiency indicator based on serum retinol concentration (< 0.7 μmol/L) (WHO, 2011) uses a cut-off aimed to identify individuals at risk of the subclinical consequences of vitamin A deficiency but had not yet developed the clinical signs associated with severe deficiency. Therefore, in their study, the vitamin prevalence values derived using serum retinol are not comparable to the estimates based on dietary intake data because the requirements criteria differ.

Unfortunately, many users of FCTs/FCDBs and vitamin A sources of requirements are not aware of the existence of two vitamin A intake definitions and/or are not aware about the differences in criteria among vitamin A recommendations and a possible mismatch of different Vitamin A definitions between intake and recommendations. Consequently, they are not aware of the impact the selection of the FCT/FCDB (with its vitamin A definition) and the source of requirement may have on vitamin A intake, requirements and adequacy level estimates. Due to the fact that many FCT/FCDB only hold RAE values and many countries use the US requirements (which are high because of the criteria used as a cut-off point), i.e., the RAE-HMD model, they may report a high apparent vitamin A inadequacy which is likely to be an overestimation. It is difficult to estimate the true vitamin A inadequacy with the actual information available. Therefore, caution should be taken when choosing the source a requirements translating vitamin A inadequacy estimations into policies and programs to combat the apparent vitamin A deficiency. Also, despite the existence of two vitamin A intake definitions and different criteria of requirements, researchers worldwide produce dietary vitamin A statistics without clearly communicating what the statistics measure and/or not mentioning the source and criteria of requirements.

### 4.2 Review of Food Composition Tables and Databases for the type of vitamin A information available

From the 90 FCTs/FCDBs reviewed, 9 % (Groups 1, 2 and 3) published total vitamin A in RE and RAE (*n* = 9), and 28 % (Group 4 with *n* = 25) allow the user to choose the system of equivalence of interest. Because so far there is no consensus on whether total vitamin A should be calculated using the system of equivalence for RE or RAE, it would be advisable that every FCT/FCDB provides data on total vitamin A in RE and RAE.

On the contrary, 43 % (Groups 8 and 9) of the FCTs/FCDBs (*n* = 38) do not provide total vitamin A or sufficient information to compute it, or there are inconsistencies between the metadata and the published values. It is surprising that a large number of FCTs/FCDBs classified in Groups 8 and 9 come from high-income countries, which are expected to possess the resources to produce high quality FCTs/FCDBs.

Poor food composition data and metadata could result in high costs to the industry and governments, and less effective policies and program interventions (Ene-Obong et al., 2019; Micha et al., 2018). On one hand, FCTs/FCDBs compilers should be aware of the critical importance of using correctly the vitamin A Tagnames, including well-documented informative metadata, and publishing total vitamin A in both RE and RAE. On the other hand, FCTs/FCDBs users must become aware of the difference between vitamin A intake definitions and be cautious when compiling vitamin A information from more than one FCT/FCDB, to avoid mixing values based on a different definition (RE and RAE). Furthermore, producers and users of dietary vitamin A inadequacy levels should select the source of requirements according to the underlying criteria that reflects the needs of the population under study and that are in line with the vitamin A definition of the intake estimation. Last but not least, publishers of journals or FCTs/FCDBs, peer reviewers and researchers should make a special effort and request to well document the definition of vitamin A intake and the source and criteria of vitamin A requirements used.

Six percent (Groups 5 and 6) of the FCTs/FCDBs (*n* = 6), provide total vitamin A in foods in either RE or RAE without providing additional information (e.g., retinol and beta-carotene equivalents) to allow the user to calculate total vitamin A using the other definition.

Finally, the remaining 13 % (group 7) of the FCTs/FCDBs (*n* = 12) do not account for the activity of alpha-carotene and beta-cryptoxanthin in their estimate of total vitamin A. This underestimation of total vitamin A in foods might induce lower estimations of dietary vitamin A consumption (especially when diets are based on fruits, vegetables, roots and tubers rich in vitamin A).

The results of this study support Melse-Boonstra and colleagues’ (2017) petition to WHO and FAO to organize an expert consultation for reviewing the evidence on the conversion of provitamin A carotenoids to retinol. We would recommend that FCTs/FCDBs publish total vitamin A in both RE and RAE. In addition, as pointed out by Dias and colleagues (2018), future changes might be expected on vitamin A definitions; therefore, it is of outmost importance that FCTs/FCDBs include the content of retinol, and the main precursors of vitamin A: beta-carotene, alpha-carotene and beta-cryptoxanthin to allow further calculations. Furthermore, they should provide the formula used to compute total vitamin A from retinol and provitamin A carotenoids.

## 5 Conclusions

Using HCES data from five countries, we found that the use of the HMD requirements keeping vitamin A consumption in μg of RAE produce a vitamin A adequacy ratio roughly three times lower than using FAO/WHO requirements keeping vitamin A consumption in μg of RE, and two times lower than using EFSA requirements keeping vitamin A consumption in μg of RE. Therefore, when planning or monitoring vitamin A policies, programs or campaigns, the selection of the vitamin A definition for intake and the source of vitamin A requirements with its underlying criteria and the corresponding unit should be carefully considered.

We have confirmed that dietary statistics of vitamin A intake in RAE and RE, as well as vitamin A indicators based on different sources of requirements, such as the vitamin A adequacy ratio, are not comparable. Therefore, vitamin A statistics and indicators used for monitoring and evaluating policies, programs and projects must be based on the same vitamin A system of equivalence for intake and source of requirements. Furthermore, when reporting on the level of inadequacy of vitamin A it is of the utmost importance to mention the source and criteria of requirements, the vitamin A definition for intake, and the FCTs/FCDBs used.

This study highlights the need for enhancing the capacity of national officers and researchers in producing, interpreting and using vitamin A statistics and indicators for designing, implementing, and/or monitoring policies and programs. Relevant and improved metadata and documentation would assist national officers to better design, implement and monitor policies and programs based on dietary vitamin A indicators and statistics.

## Author statement

**Ana Moltedo:** Conceptualization, Methodology, Software, Investigation, Formal Analysis, Writing - Original Draft, Visualization. **Cristina Álvarez-Sánchez:** Conceptualization, Methodology, Investigation, Validation, Visualization, Writing - Review & Editing. **Fernanda Grande:** Visualization, Writing - Review & Editing. **U. Ruth Charrondiere:** Writing - Review & Editing.

## Declaration of Competing Interest

None.
